# Green Endoscopy: A Review of Global Perspectives on Environmental Sustainability of Gastrointestinal Endoscopy

**DOI:** 10.3390/jcm14113936

**Published:** 2025-06-03

**Authors:** Adishwar Rao, Abdullah Sultany, Amlish Gondal, Raja Chandra Chakinala, Hareesha Rishab Bharadwaj, Saurabh Chandan, Hassam Ali, Sheza Malik, Saqr Alsakarneh, Dushyant Singh Dahiya

**Affiliations:** 1 Department of Internal Medicine, Guthrie Robert Packer Hospital, Sayre, PA 18840, USA; abdullah.sultany@guthrie.org (A.S.); amlishbgondal@gmail.com (A.G.); 2 Department of Gastroenterology, Guthrie Robert Packer Hospital, Sayre, PA 18840, USA; rajachandra@gmail.com; 3 Faculty of Biology Medicine and Health, The University of Manchester, Manchester M13 9PL, UK; rishabbharadwaj31@gmail.com; 4 Center for Interventional Endoscopy (CIE), Advent Health, Orlando, FL 32803, USA; saurabhchandan@gmail.com; 5 Advanced Endoscopy, Houston Methodist West Hospital, Houston, TX 77094, USA; 6 Department of Gastroenterology, Hepatology & Nutrition, ECU Health Medical Center/Brody School of Medicine, Greenville, NC 27834, USA; hassamali155@gmail.com; 7 Department of Internal Medicine, Rochester General Hospital, Rochester, NY 14621, USA; sheza.malik683@gmail.com; 8 Department of Internal Medicine, University of Missouri-Kansas City, Kansas City, MO 64110, USA; s.alsakarneh@umkc.edu; 9 Division of Gastroenterology, Hepatology & Motility, The University of Kansas School of Medicine, Kansas City, KS 66103, USA; dush.dahiya@gmail.com

**Keywords:** green endoscopy, sustainable endoscopy, carbon footprint, greenhouse gases, emissions

## Abstract

Endoscopic procedures are the cornerstone of intervention in gastroenterology—from evaluating common illnesses to non-surgically managing complex diseases. Expectedly, these procedures are linked to greenhouse gas (GHG) emissions globally and contribute significantly to the global climate change crisis. Professional gastroenterology societies globally raise awareness of this evolving crisis and suggest specific measures to appropriately measure the burden contributed by endoscopy units and mitigate the environmental impact of this common clinical practice. To the unsuspecting eye, the solution to this crisis is relatively simple: decrease the utilization of endoscopic procedures. However, the dependence of modern medicine on these procedures, both diagnostically and therapeutically, makes it significantly more challenging to reduce their utilization. Instead, a structured approach to systematically consider the specific indications for each procedure, minimize waste generation, promote recycling of waste products, and limit the number of repeat endoscopies until clinically necessary may be more pragmatic to reduce GHG emissions globally. In this narrative review, we discuss the perspectives of global gastroenterology societies on sustainable or “green” endoscopy and summarize their recommendations to aid the day-to-day gastroenterologist in making their contribution to environmental sustainability while providing optimal care to their patients.

## 1. Introduction

The present-day healthcare sector contributes uniquely to the ongoing global warming crisis [1]. On one end, increasing focus on enhancing patient care, comprising stricter infection control and the use of non-reusable equipment, has improved patient outcomes; on the other end, it has also contributed to an increase in carbon footprints [2,3]. The global average carbon footprint per person is approximately 5 tons; however, in the United States (US), these estimates may be over three times the global average [4,5]. Nearly 4% of the global greenhouse gas emissions (GHGEs) can be attributed to the healthcare sector, while this number may be as high as 8% in the US [6,7,8,9]. Notably, healthcare without harm can directly or indirectly contribute more than 70% to the healthcare sector’s global carbon footprint [10]. Surgical specialties and other procedure-intense non-surgical specialties, such as gastroenterology, cardiology, and critical care medicine, require a significant amount of instrumentation for the smooth facilitation of the standard of care for patients and, therefore, are often more predisposed to waste generation than other nonprocedural specialties [11,12].

Gastrointestinal (GI) endoscopy ranks among the top three hazardous waste-generating medical interventions, contributing approximately three kilograms of waste per bed in a day [13]. GI endoscopy units are essential to the preventative, diagnostic, and therapeutic workforce for patients affected by GI illnesses. Estimates suggest that in the US alone, almost 18 million endoscopies are performed yearly [6,11]. Using highly specialized devices to anatomically decipher these illnesses inevitably predisposes gastroenterologists to use disposable materials significantly. It was not until a few years ago that the estimates for endoscopic procedural waste were attempted to be calculated after audits frequently pointed towards a high waste burden with these procedures [6,14]. Each year, the total endoscopic waste in the US alone is over 40,000 tons. Nearly two-thirds of this waste gets labeled, appropriately or inappropriately, as “biohazard”, which requires specific procedures to be disposed of adequately [4,6].

Meanwhile, an increasing focus on the sustainability of healthcare practices and lowering the industry’s carbon footprint has contributed to introducing “green endoscopy or sustainable endoscopy”, an approach to minimize the environmental impact of endoscopic procedures [15]. This approach covers most aspects of endoscopies, ranging from clinical to logistic aspects, to understand the areas corresponding to significant GHGEs, address factors which contribute to this environmental insult, promote international inter-institutional collaboration, and educate healthcare workers (HCWs) on more judicious utilization of the available resources while ensuring that each patient receives optimal clinical care.

We searched indexing databases, such as PubMed, Google Scholar, and Scopus, using the search terms “green endoscopy”, “sustainable endoscopy”, “carbon footprint in gastrointestinal endoscopy”, and “emissions related to gastrointestinal endoscopy” to select articles for this narrative review. A further selection of articles also included bibliographies of the previously selected articles. The literature was screened based on relevance, recency, and clinical appropriateness for sustainable endoscopy. A time frame did not bind the search, and only the literature published in English was selected. Hence, in this review, we discuss the concept and principles of green endoscopy, current global perspectives, and its applicability amidst the current endoscopic practices.

## 2. Environmental and Digestive Health Impact of GI-Related Emissions

The carbon footprint represents GHGEs in terms of carbon dioxide equivalents [16]. GHGEs contribute to climate change, disturb the ecological balance of the environment, cause weather fluctuations, and alter the dynamics of infectious vectors [17,18]. These contribute to an alteration in the incidence and prevalence of GI infections and autoimmune conditions. Moreover, they may also disturb the gut–brain physiology and lead to malnutrition. Nutritional imbalances can affect the prevalence of metabolic liver disease, while phenomena such as flooding due to climate change can increase the risk of infectious hepatitis and toxic liver disease [19]. The impact of GI-related GHGEs on the environment is, therefore, heavily intertwined with the effects of climate change on digestive health [20]. Consequently, the excess healthcare burden generated by the aforementioned factors requires additional resource utilization, including manufacturing and transporting medications and medical equipment. It also requires greater human resource mobilization and utilization and, in turn, increases the healthcare sector’s economic liability.

GHGEs from endoscopic units can broadly be classified into three scopes for segregation and understanding. Scope 1 emissions are direct emissions from factors such as carbon dioxide used for insufflation in endoscopic units and transportation for healthcare professionals and patients. Scope 2 emissions are indirect emissions and represent the utilization of fossil fuels to generate energy sources for the routine functioning of endoscopy units. Scope 3 emissions are indirect and comprise the supply chain and waste-related emissions, accounting for approximately 70% of GHGEs [4]. While it is challenging to quantify the environmental burden from each scope of emission accurately, estimates suggest that a hundred endoscopies have the potential to generate over 300 kgs of solid waste and nearly 1400 gallons of liquid waste, in addition to approximately consuming 2000 kW-h of energy [14]. Furthermore, a significant proportion of the generated waste is non-recyclable plastic, which can be degraded into microplastics. Microplastics can be ingested, have deleterious effects on human health, and have been previously isolated from human colectomy specimens [21].

## 3. Sustainable Endoscopy Principles

**Figure 1:**
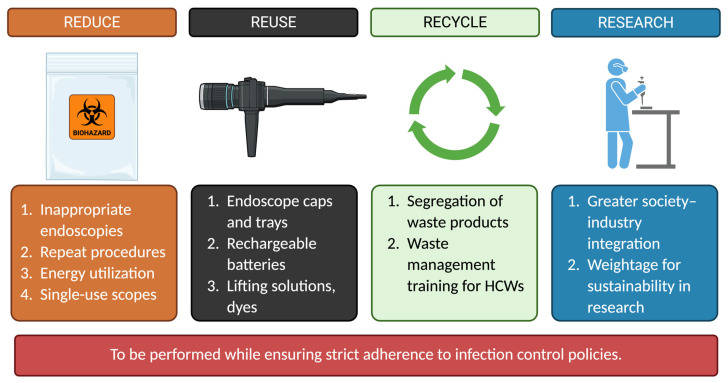
Principles of sustainable endoscopy with suggestions for implementation. HCWs, healthcare workers. Figure created with BioRender [27].

A broad global consensus favors attenuating the environmental footprint of endoscopic procedures while ensuring economic feasibility and regulatory compliance [22,23,24]. The integral principles of sustainable endoscopy depict multifactorial carbon footprint control, ranging from source control to recycling objects. “Reduce, reuse, recycle, research” is a motto that encompasses these sustainable endoscopy principles (Figure 1). “Reduce” comprises promoting source control of the carbon footprint by ensuring the appropriateness of each endoscopic intervention and histologic analysis, limiting the number of repeat procedures, avoiding the use of single-use endoscopes, as feasible, and utilizing energy-consuming resources judiciously, while ensuring compliance with best practices [25]. It is worth noting that a multicenter study found that nearly 30% of endoscopy referrals were inappropriate based on the American Society of Gastrointestinal Endoscopy (ASGE) recommendations, with most of these referrals being for surveillance [26].

“Reuse” encourages reusing any instrumentation not used during a procedure at a later event, using rechargeable batteries, and sharing unused solutions with other endoscopy suites, as deemed appropriate. “Recycle” promotes the segregation of waste products and staff training on waste management, as needed, to recycle waste products appropriately, even though the use of recycled goods may be limited in the healthcare setting. Additionally, increasing focus on “research” as another component of sustainability promotes the computation of statistics relating to the healthcare sector’s environmental burden and exploration of specific indications of single-use (SU) equipment to limit unnecessary carbon emissions in disposing of the SU equipment. It also propagates these indications educationally by collaborating with GI societies to limit our carbon footprint [23,24,25].

### 3.1. Current Global Perspectives on Green Endoscopy

Major gastroenterology societies globally, including the European Society of Gastrointestinal Endoscopy (ESGE), European Society of Gastroenterology and Endoscopy Nurses and Associates (ESGENA), British Society of Gastroenterology (BSG), ASGE, Asia Pacific Association of Gastroenterology (APAGE), Italian Association of Hospital Gastroenterologists and Digestive Endoscopists (AIGO), Joint Advisory Group (JAG), and Center for Sustainable Health (CSH), note the importance of sustainable endoscopic practices [22,23,24,28]. Moreover, four major GI societies in the US, comprising the American College of Gastroenterology (ACG), American Gastroenterological Association (AGA), American Association for the Study of Liver Diseases (AASLD), and ASGE, have developed a 5-year multi-society strategic plan to attenuate the bidirectional impact of climate change on digestive health, and vice versa [20].

Beginning in the year 2023 and planned until 2027, this multi-society strategic plan aims first to assess the environmental impact of GI-related emissions, develop policies across societies, and engage the healthcare industry to promote sustainability and then monitor the impact of their work and integrate sustainability as an indicator of the quality of care provided to patients [20]. The first and foremost consideration should be rationalizing the decision to perform an endoscopy if other less invasive modalities are available without considerable differences in the testing sensitivity/specificity.

As an example, for low-risk lesions across the GI tract, such as an inlet patch in the esophagus, Los Angeles grade A or B erosive esophagitis, unifocal intestinal metaplasia of the stomach or atrophic gastritis without dysplasia, duodenal peptic ulcer, or low-risk colonic adenomas, it may be worthwhile reconsidering endoscopic surveillance, both in terms of patient comfort and economic viability and environmental sustainability. Similarly, the urea breath test has comparable testing utility over endoscopy in the diagnosis and eradication of Helicobacter pylori [24,29]. Serum and fecal biomarkers, such as c-reactive protein and calprotectin, may also help exclude inflammatory bowel disease (IBD) in patients with low overall clinical suspicion for IBD who present with irritable bowel features [30,31]. Gastroenterologists should, therefore, refrain from performing endoscopies without a distinct indication and avoid doing so in borderline indications [30]. While a significant proportion of diagnostic evaluations are standardized and guideline-based in the current era, societal recommendations should incorporate their environmental sustainability in addition to their clinical utility. The ecological impact of surveillance endoscopies may go unnoticed unless a keen eye is kept out for it.

Surveillance endoscopies typically involve protocol-driven biopsies, culminating in multiple biopsies being obtained and then transferred for histopathologic assessment in separate containers, which may lead to a waste of resources. For example, the Seattle protocol for Barrett’s Esophagus (BE) recommends performing four-quadrant biopsies for the segment at intervals of 2 cm or less [32,33]. While this is the gold standard for identifying even subtle dysplasia as per the ASGE recommendations for the optimal and early diagnosis of esophageal adenocarcinoma, there does appear to be a need for improved techniques that require lesser resource utilization while maintaining the sensitivity and specificity of the modality. Although not currently endorsed as a gold-standard equivalent by the ASGE, virtual chromoendoscopy does have a reasonable mention in their most recent guideline to improve diagnostic accuracy for esophageal cancer in patients with BE [33]. Using artificial intelligence (AI) as an adjunct to identify dysplastic mucosal regions in conditions such as BE may be another environmentally sustainable alternative to limit healthcare resource wastage [23,24].

The use of SU endoscopes constitutes a significant concern for sustainability in the clinical domain of gastroenterology. SU duodenoscopes were introduced into mainstream clinical practice after reports of infections with multidrug-resistant organisms surfaced in patients undergoing endoscopic retrograde cholangiopancreatography (ERCP) [34,35]. As the specialty evolved, further evidence revealed that inadequate reprocessing of traditional duodenoscopes may have contributed to harboring these infectious agents. However, it is particularly challenging to evaluate infectious outcomes between SU duodenoscopes and regular use (RU) duodenoscopes because of the overall low rates of infection with both types of scopes, necessitating an inadequately large sample size and, in turn, creating a logistical research nightmare [23,36,37].

Life cycle assessments (LCAs) have demonstrated that ERCP performed with SU scopes is associated with at least 20–40 times greater carbon dioxide emissions than RU scopes, a difference primarily attributed to terminal incineration emissions for the disposal of SU scopes [23,38]. Economic viability for preferring either of these scopes remains to be explored in depth, with some evidence suggesting that scopes with specific disposable components, such as disposable end caps, may strike an appropriate balance between infection stewardship, economic viability, and logistic convenience [39]. Interestingly, a web-based survey of over 400 ESGE and ESGENA members conducted by the ESGE noted that a majority of SU endoscope users believed that their institution did not have reprocessing capabilities for endoscopes. Moreover, inadequate policy support, staff unawareness, costs, and a lack of prioritization of sustainable endoscopy emerged as major barriers towards sustainable endoscopy [40].

Optimizing logistic factors can considerably influence the environmental impact of endoscopies. It can range from changes in scheduling to more appropriate waste disposal techniques. Endoscopies should preferably be performed on an outpatient basis to avoid overnight hospital stays and unnecessary utilization of healthcare resources, unless clinically contraindicated. If clinically appropriate, bidirectional endoscopies should be performed in the same session. Similarly, endoscopic ultrasound and ERCP should also be attempted in the same session to reduce disposable equipment utilization and operational and staffing costs [23]. Periprocedurally, endoscopists should consider being mindful of long-lasting environmental impacts of easily overlooked factors, such as water use during endoscopic decontamination, flushes, immersion colonoscopy, and automated flushing systems [22].

Standard operating procedures on periprocedural water utilization may be environmentally beneficial, such as using tap or filtered water flushes instead of sterile water and using manual flushes over automated ones [41,42]. Stringent compliance with hospital and society-guided infection control protocols while making these modifications is a comprehended principle. Optimal waste disposal methods, using the principles of sustainable endoscopy and limiting the non-essential utilization of non-medical resources, may aid in limiting periprocedural environmental hazards. Judicious use and disposal of PPE and scrubs, provisioning motion sensing lights or turning off lights when not needed, and limiting non-essential commutes to the workplace may contribute to environmental sustainability. It is worth noting that the setup for lighting in the endoscopy suite can consume more energy than the endoscopic apparatus itself. Interestingly, something as minor as switching from traditional halide bulbs to light-emitting diode bulbs in the endoscopy suites may reduce energy utilization by approximately 60% [43].

A Japanese prospective study evaluated the utility of using isolation gowns as a part of personal protective equipment (PPE) during endoscopies by assessing the growth of microorganisms on various parts of the gown after endoscopic procedures using the stamp method. Contamination rates for gowns ranged from 30 to 77%, depending on operator experience, with the minimum attributed to expert endoscopists and the maximum attributed to resident physicians. Interestingly, cultures obtained from contaminated isolation gowns only comprised non-pathogenic bacteria from the tap water used during the procedure and from the patient’s skin or mouth flora. While the study only examined a few patients undergoing upper GI endoscopy, it may be worthwhile to investigate the relevance of changing isolation gowns after each upper GI endoscopic procedure [44].

Furthermore, greater collaboration between the healthcare sector and the industry may open doors to modified endoscopic designs, such as reloadable endo-clips and modified band ligators [45,46]. Similarly, the architectural sustainability of endoscopic units may be worth considering when designing or remodeling endoscopic units. Lastly, to ensure environmental sustainability without compromising patient care, it is imperative to include principles of green or sustainable endoscopy into the training curricula for gastroenterology fellows and endoscopy unit staff and as a continued medical education activity for practicing gastroenterologists [47]. Moreover, sustainability and preventative gastroenterology should be intertwined to target source control despite its limitations in resource-constrained settings.

Interventions directed toward sustainability should not only be environmentally favorable but also economically viable and clinically feasible (Table 1). Interventions with low overall feasibility involve either a significant economic burden, specialized equipment and training, or a heavy dependence on regulatory approvals and device availability through the industry. Those with moderate feasibility may depend on situational factors and local hospital circumstances, such as clinical indications and scheduling logistics. Highly feasible interventions include interventions that rely heavily on appropriate clinical judgement, have adequate logistic coherence (such as endoscope reprocessing units), are easily implementable, require mere educational or behavioral interventions, and do not significantly disrupt the workflow. The applicability and utility of these environment-favoring interventions in lower- and middle-income countries (LMICs) significantly depend on their economic viability and feasibility. Feasible interventions that are also economically viable would be preferentially applicable in LMICs.

### 3.2. Overcoming Challenges to a Greener Future

A significant challenge to sustainability is the limited awareness among HCWs in day-to-day clinical gastroenterology practice. There is a dearth of high-quality epidemiologic data on the prevalence of unawareness; however, limited survey data suggests that the awareness about green endoscopy in HCWs may be as low as 16% [48]. Encouragingly, over 65% of the participants from the same survey endorsed their willingness to participate in green endoscopy campaigns. Provisions for adequate segregation of research funding, incorporation of sustainability into the training curriculum of not just HCWs but also graduate and undergraduate students, inculcation of a mindset of sustainability instead of playing catch-up in the long run, and setting global standards for sustainability in gastroenterology may help overcome multiple barriers towards sustainable endoscopy [49].

A prospective study at a large tertiary hospital in the US included patients undergoing surveillance colonoscopy with a Boston Bowel Preparation Scale score of seven or more. Their intervention comprised a discussion of sustainable endoscopy practices with gastroenterologists, then evaluating the number of tools utilized during the colonoscopies. At the end of 14 weeks of the study, they computed the frequency of utilization of a single tool or multiple tools, with the use of multiple tools considered environmentally deleterious. Single-tool, i.e., biopsy forceps or snare, utilization was significantly higher in the intervention group than in the non-intervention group (49% vs. 32%, *p* = 0.003). The study also showed a significant reduction in the usage of multiple tools after the intervention (17% vs. 33%, *p* = 0.002) [50]. Furthermore, evidence also indicates the feasibility of one-device colonoscopies towards environmental sustainability [51].

Another challenge to sustainability is inappropriate waste segregation [52]. Periprocedural waste can be segregated into direct landfill waste (DLW), regulated medical waste (RMW), and recyclable waste (plastic and paper waste). Approximately 50–65% of the generated endoscopic waste can be DLW, 30–40% RMW, and the rest recyclable waste [6,53]. Neves JA et al. conducted a prospective interventional study to evaluate the impact of behavioral interventions on waste segregation and eventual reduction in endoscopy-related emissions. They conducted seminars for all HCWs involved in the practice of endoscopy [53]. When comparing the waste generation and total carbon footprint (TCF) before and 1 month after the intervention, in terms of kilograms of carbon dioxide emissions, there was a statistically significant reduction in RMW (mean RMW: ~362 kg vs. ~212 kg, *p* = 0.010) and the TCF (mean TCF: ~439 kg vs. ~300 kg, *p* = 0.018). Notably, there was no increase in emissions 4 months after the intervention compared to 1 month, which is an encouraging sign about the longevity of relatively simple behavioral interventions.

### 3.3. Role of Technology in Sustainability

Traditional white light endoscopy is often less suited to detect subtle lesions and early-stage malignancies, potentially requiring more frequent confirmatory biopsies for such lesions. To tackle this, image-enhanced endoscopy, which uses techniques such as narrow band imaging-magnifying endoscopy (NBI-ME), blue laser imaging, or linked color imaging to assess mucosal surfaces more accurately, may be the more environmentally sustainable alternative. The sensitivity and specificity of NBI-ME can be greater than 90% in detecting neoplastic upper and lower GI lesions [54]. Similarly, capillary patterns noted on NBI-ME could help evaluate atypia in colorectal neoplasms in their early stages [55]. This could reduce the number of biopsies required, ease the entire logistical chain involved in performing histopathological analysis of these samples, and limit the resources used for their waste disposal.

The role of technology does not necessarily revolve around direct innovation and implementation into clinical gastroenterology. Technology is also vital to maintaining supply chains of medical equipment and instruments used in day-to-day GI practice. Currently, in the majority of high-income nations, linear supply chains are heavily relied upon due to a relative abundance of resources and finances [56]. This means that manufactured goods eventually terminate as healthcare waste and are rarely reused, which has, understandably, been a practice to mitigate the infectious burden in gastroenterology. Given the increasing focus on sustainability, a transition to a more closed-loop supply structure should be strenuously considered, where at least a part of the manufactured goods is reused in the healthcare setting or, if not suitable for clinical use, in the non-healthcare setting.

Lastly, when discussing technology, it is imperative that we utilize it most appropriately, not only for equipment and techniques but also for improving sustainability in the academic gastroenterology world. A small Canadian study estimated the carbon costs of traveling to attend a gastroenterology conference in the country [57]. Even though they excluded international attendees, strikingly, the average carbon burden per attendee was over 200 tons or 500 kgs of carbon dioxide. To understand this better, each kg of carbon dioxide nearly equals 600 L of carbon dioxide gas, and this emission can be compared to the emissions from routinely driving a car for approximately two months in the same region [57]. Physical presence at major international conferences may thus unintentionally contribute to global environmental deterioration. Therefore, encouraging the use of technology to conduct these conferences, or at least a part of them, virtually, may benefit the environment. As academic intellectuals across the globe, we should encourage our societies to lead and pioneer environmental sustainability in gastroenterology and introspect on reducing wasteful emissions to reduce environmental burdens as well.

### 3.4. Endoscopy in Specific Settings

Diagnostic endoscopies form a significant proportion of our discussion on sustainable endoscopy, mainly due to the potential corrective impact attributable primarily to the sheer volume of these procedures. Notably, colorectal cancer (CRC) is one of the most prevalent and lethal cancers in the world, which makes the use of screening methodologies indispensable [58,59]. Newer endoscopic techniques, such as colon capsule endoscopy (CCE), add to the preexisting non- or minimally invasive body of CRC testing modalities, including fecal immunochemical testing and computed tomographic colonography. The sensitivity and specificity of CCE may be as high as 87% each for polyps that are 6 mm or less in size and 87% and 95%, respectively, for polyps that are 10 mm or less in size, based on pooled estimates from studies [58,60,61]. Therefore, although not the gold standard, CCE can be discussed as an alternative for CRC screening in patients unwilling to undergo colonoscopy as part of shared decision-making or in whom colonoscopy is contraindicated [58,61].

While CCE appears to offer a more sustainable approach to CRC screening than colonoscopy, its numerous limitations must be addressed thoughtfully when deciding on a testing modality for patients. These capsules are complex instruments and comprise multiple working components. The precise estimates of carbon dioxide emitted in manufacturing these capsules (or their parts) are unclear, although it may be reasonable to assume that it is not low. Moreover, these capsules are, again, single-use and only provide diagnostic benefits, while colonoscopes can be used to therapeutic advantage. On the contrary, the upsides of CCEs cannot be overlooked either. CCEs generally require less medical instrumentation overall and disposal than traditional colonoscopies. Additionally, carbon dioxide insufflation, often preferable over air insufflation in conventional colonoscopies, is not required in CCE, thereby eliminating direct procedural emissions. However, given the lack of empirical evidence favoring CCEs regarding sustainability, they cannot be broadly categorized as environmentally superior, although they can be considered on a case-by-case basis where CRC screening may be required, but colonoscopy is not an alternative due to contraindications or patient preference [58].

Although diagnostic endoscopies comprise a major section of routine GI endoscopies, evaluating the carbon footprint of therapeutic endoscopic procedures in specific endoscopic settings is worthwhile. A post hoc LCA of the “RESECT-COLON” trial comparing the environmental impact of endoscopic submucosal dissection (ESD) and piecemeal endoscopic mucosal resection (PEMR) for large colonic adenomas suggested nearly 18% greater emissions in patients undergoing PEMR and its first follow-up at an expert center, with subsequent follow-ups at local centers [62,63]. However, the same study’s scenario simulation also revealed that if PEMR could be performed and followed locally while upholding adequate clinical standards and quality checks, it may offer a nearly 10% reduction in carbon emissions compared to ESD, which needs to be performed and initially followed up at an expert center.

## 4. Expert Commentary

The growing importance of sustainability in the healthcare sector elevated our responsibility to balance clinical outcomes, patient safety, and environmental sustainability. Suffice it to say that with the current evidence, the utility of small and relatively easy behavioral interventions to reduce emissions is significant in GI endoscopy. However, before implementing more clinical and practice-altering interventions, such as preferentially using reusable endoscopes, modifying periprocedural tactics, and using AI for routine surveillance, epidemiologically credible interventional studies are needed to avoid diagnostic compromise and reduced procedural efficiency. Moreover, we cannot overlook additional barriers, such as financial constraints, particularly in LMICs, and policy-level resistance to sustainability. A reasonable workaround would be to ascertain sustainable interventions that are economically favorable as well. This can only be accomplished through intertwined efforts between HCWs, industrialists, and policymakers. We envision sustainability firmly embedded into the fabric of clinical GI practice in the near future, a task that seems herculean to the lay eyes but faces a community growing like no other due to its resilience.

Targeting Scope 3 emissions as an initial step in sustainability may be a reasonable start [64]. To build on that and achieve over 50% reductions in GHGEs, there needs to be synchrony between instrument suppliers and the consumers, i.e., manufacturing and supply chain, hospitals, and endoscopy units. Taking small, sustained steps is the key to reliable progress in environmental sustainability and is attainable through culture change in conjunction with technological and clinical procedural innovation. Additionally, given the encouraging current evidence on the efficacy of using relatively simple behavioral interventions, we must emphasize sustainability as a global priority. Streamlining diagnostics through improved collaborative frameworks and AI for surveillance endoscopies can reduce unnecessary interventions, remove waste, and improve efficiency.

## 5. Conclusions

Gastrointestinal endoscopy is one of the most significant contributors to the global carbon footprint. While we have made strides in improving the clinical management of our patients, there are lacunae in our understanding of the environmental impact of this progress. In this narrative review, we summarized the global perspectives on sustainability in endoscopy and how each health professional can contribute, even with minimal effort, to ensure a greener tomorrow for future generations. Superior endoscopic stewardship, adherence to society and local hospital guidelines, and incorporation of technology and AI into routine clinical practice while limiting unnecessary commutes to the workplace and optimizing the scheduling of procedures may be the way to move forward.

Overcoming critical challenges, including a lack of awareness and constrained resources, is vital in aligning sustainability with the best patient care. By making small changes today, there is an opportunity to build a more sustainable global health system for tomorrow. The road to a greener gastroenterology practice surely has challenges that must be encountered with a deep commitment to excellent patient care and extensive multidisciplinary and multi-society collaborations. Despite the daunting challenges ahead of us, the future of green endoscopy looks encouraging.

## Figures and Tables

**Table 1:** Comparative environmental impact, economic viability, feasibility, and applicability of various interventions.

Intervention	Environmental Impact	Economic Viability	Feasibility	Resource Accessibility-Based Applicability
Clinical appropriateness	Lower energy wastage due to procedural volume stewardship.	High	High	Universal
Bundling two or more procedures	Lower human resource and endoscopic suite utilization per patient.	Moderate	Moderate	Resource-abundant
Advanced imaging and artificial intelligence	Higher upfront manufacturing energy expenditure but lower downstream logistical utilization.	Low	Low	Resource-abundant
Use of reusable equipment	Potential avoidance of emissions during the disposal of single-use equipment.	Moderate	High	Resource-limited
Waste sorting and recycling	Appropriate segregation can reduce disposal emissions substantially.	High	High	Universal
Water conservation	Low-risk, high-yield energy conservation strategy.	High	High	Resource-limited
Energy/resource efficiency	Low-risk, high-yield modifications like motion-sensing lights support sustainability and energy efficiency.	Moderate	High	Universal
Innovative device design	Lower per-procedure wastage.	Moderate	Low	Resource-abundant
Human resource development (education, training)	Directly or indirectly drives all the above interventions.	High	High	Universal
